# Ramsay Hunt Syndrome Associated with Central Nervous System Involvement in an Adult

**DOI:** 10.1155/2016/9859816

**Published:** 2016-04-13

**Authors:** Tommy L. H. Chan, Ana M. Cartagena, Anne Marie Bombassaro, Seyed M. Hosseini-Moghaddam

**Affiliations:** ^1^Department of Clinical Neurological Sciences, London Health Sciences Centre (LHSC), London, ON, Canada; ^2^Pharmacy Services, London Health Sciences Centre (LHSC) and Department of Medicine, Schulich School of Medicine and Dentistry, Western University, London, ON, Canada; ^3^Division of Infectious Diseases, Department of Medicine, London Health Sciences Centre (LHSC), London, ON, Canada

## Abstract

Ramsay Hunt syndrome associated with varicella zoster virus reactivation affecting the central nervous system is rare. We describe a 55-year-old diabetic female who presented with gait ataxia, right peripheral facial palsy, and painful vesicular lesions involving her right ear. Later, she developed dysmetria, fluctuating diplopia, and dysarthria. Varicella zoster virus was detected in the cerebrospinal fluid by polymerase chain reaction. She was diagnosed with Ramsay Hunt syndrome associated with spread to the central nervous system. Her facial palsy completely resolved within 48 hours of treatment with intravenous acyclovir 10 mg/kg every 8 hours. However, cerebellar symptoms did not improve until a tapering course of steroid therapy was initiated.

## 1. Case Presentation

A 55-year-old female presented to the emergency department with acute intermittent unsteadiness worsening with mobility. Her initial workup including brain computed tomography (CT) scan was interpreted as normal and she was discharged home. Three days later, her unsteadiness progressed and she developed a new onset of right ear pain and diplopia. She returned to the emergency department and neurological examination revealed right ear erythematous vesicular lesions, gait ataxia, limb dysmetria, right-sided facial palsy, and fluctuating horizontal diplopia. She denied fever, headache, nuchal rigidity, altered level of consciousness, vertigo, weakness, or any other neurological complaints.

Her medical history included well-controlled type 2 diabetes, hypertension, and hyperlipidemia. Medications included metformin, ramipril, metoprolol, atorvastatin, and acetylsalicylic acid.

Due to the new onset of neurological signs and symptoms, a repeat brain CT scan was performed followed by lumbar puncture (LP). Brain CT was interpreted as normal. Cerebrospinal fluid (CSF) revealed elevation in glucose, nucleated cells (lymphocyte-dominant), and protein (Table 1).

The patient was initially treated empirically with intravenous broad-spectrum antimicrobial therapy including ceftriaxone 2 g every 12 hours, vancomycin 15 mg/kg every 12 hours, and acyclovir 10 mg/kg every 8 hours. Subsequently, varicella zoster virus (VZV) was detected in CSF by polymerase chain reaction (PCR) and antibiotics were discontinued. She had no history of chicken pox or vaccination against VZV. One day after presentation, follow-up contrast brain magnetic resonance imaging (MRI) showed nonspecific cerebral deep white matter hyperintensities likely due to chronic small vessel changes.

The facial palsy completely resolved within 48 hours of starting intravenous acyclovir and the right ear edema improved dramatically; however, she continued to have gait ataxia, dysmetria, fluctuating diplopia, mild right ear hearing deficiency, and vesicular lesions. She remained in the hospital for 7 days and was discharged on intravenous acyclovir to complete a total 21-day course at a dose of 10 mg/kg every 8 hours.

Upon follow-up, the patient was found to have ongoing cerebellar findings, including dysarthria, dysmetria, gait ataxia, and inability to perform the tandem walk test. The remainder of her neurologic examination including cognitive testing was normal. Laboratory results revealed a normal C-reactive protein (CRP) of 2.5 mg/L and white blood cell count (WBC) of 8.9 X 10^9^/L, but the erythrocyte sedimentation rate (ESR) was mildly elevated at 33 mm/h. A tapering course of high dose prednisone (60 mg initially and 10 mg taper weekly for a total of 6 weeks) was added to the treatment regimen due to her ongoing neurological signs.

**Figure 1:**
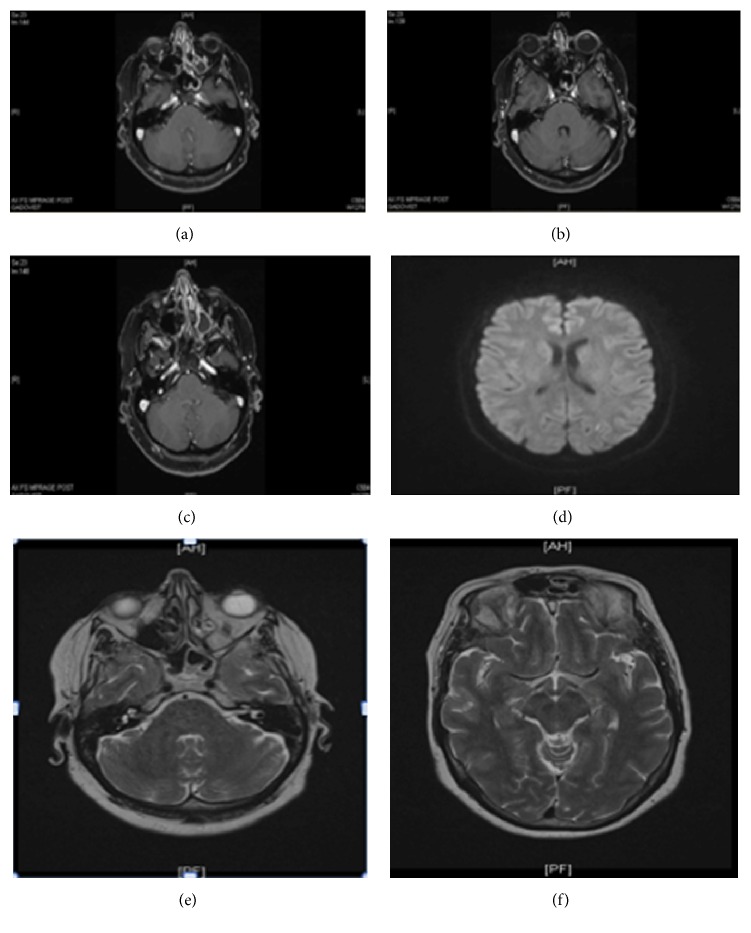
Brain magnetic resonance imaging (MRI) with contrast 6 weeks later. (a, b) T1 (magnetization-prepared rapid acquisition with gradient echo): tiny focus of enhancement seen within the superior portions of the right internal auditory canal. (c) T1 (magnetization-prepared rapid acquisition with gradient echo): tiny focus of enhancement seen at the posterior portions of the basal turn of the cochlea. (d) Diffusion Weighted Image: left superior occipital white matter with restricted diffusion. (e) T2-weighted sequence: no enhancement at the cerebellum and pons. (f) T2-weighted sequence: no enhancement at the midbrain.

Her neurological examination 6 weeks later showed a significant improvement (Figure 1); however, she had recurrence of right ear vesicular lesions without central nervous system (CNS) involvement; therefore, oral acyclovir 800 mg three times a day was prescribed for five additional days.

## 2. Discussion

VZV is a human herpes virus and the primary infection causes varicella (chicken pox) [1]. After initial infection, the virus lies dormant in cranial nerve ganglia, dorsal root ganglia, and autonomic ganglia [1]. Typically, cell-mediated immunity suppresses the replication of the virus. However, reactivation of the virus to cause herpes zoster (shingles) and potential neurological complications may occur, especially in the immunocompromised host [2].

Ramsay Hunt syndrome (RHS), also called herpes zoster oticus (HZO), is an example of reactivation of the VZV specifically at the geniculate ganglion. It classically consists of the triad of ipsilateral facial paralysis, ear pain, and vesicular lesions in the auditory canal and auricle [3]. The course of illness may vary depending on the extent of the infection and timing of therapy [4]. Complications include CNS involvement and irreversible nerve damage [2].

We describe an adult patient who developed RHS with CNS involvement, particularly of the cerebellum. Our patient presented with gait ataxia followed by painful erythematous vesicular lesions in the right ear. She later developed transient right facial palsy, dysmetria, dysarthria, and fluctuating diplopia. These findings were highly suggestive of cerebellar dysfunction. The diplopia suggested possible brainstem involvement as well. Clinical signs, symptoms, CSF pleocytosis with increased protein, and detection of VZV in the CSF by PCR confirmed CNS involvement. Acute cerebellar ataxia following VZV infection is a common neurological complication in children; however, it is extremely rare in adults [4, 5]. A PubMed literature search limited to the English language and adults until February 2015 revealed only 2 cases of RHS complicated by cerebellitis [6, 7]. An additional 3 reports described brainstem involvement [8–10]. Only two of these 5 cases involved immunocompetent adults with PCR confirmation of VZV in the CSF [6, 9] Similar to our case, both of these patients were females with one of them having well-controlled diabetes. The coexistence of RHS and VZV encephalitis has also been rarely documented in the English-language literature [11–13]. The mechanism of VZV cerebellar encephalitis is unclear, but hematogenous spread or dissemination through the CSF pathways has been hypothesized [7]. Immunosuppressants have been shown to impair leukocyte migration into the brain, which could explain why immunocompromised patients are at higher risk of VZV dissemination to the CNS [12].

Early diagnosis and management improve prognosis in RHS [14]. The diagnosis of RHS is based on patient history and clinical examination. Our patient presented with unsteadiness and the appearance of erythematous vesicular lesions 3 days later. It has been reported that the onset of RHS may not occur simultaneously with the eruption of vesicular lesions [15]. The diagnostic and prognostic value of radiographic and CSF analysis has not been determined [16]. However, in this report, we demonstrated the utility of both investigations. The acute to subacute small white matter infarct in the left superior occipital white matter and the narrowing of the 4th portion of the posterior cerebral artery (PCA) suggested possible cerebral angiitis associated with VZV encephalitis; however, the gold standard in terms of diagnosing cerebral vasculitis is digital subtraction angiography (DSA) and biopsy. In fact, RHS with VZV vasculopathy has been previously discussed [17]. Stroke and transient ischemic attacks due to viral infection of blood vessels are not uncommon [18]. Our patient exhibited radiographic evidence of stroke and arterial narrowing in the absence of clinical symptoms. Conversely, the cerebellar and brainstem clinical signs did not correlate with the radiographic findings. Thus, the possibility of CNS involvement should not be excluded based on the absence of radiograph findings. Given that our patient had improved clinically, further investigation was deemed unnecessary.

The prognosis for facial palsy is poor in RHS: only 10% of patients will have complete resolution of their facial palsy despite treatment [15]. Antivirals, such as acyclovir, are commonly used to improve the chance of recovery of facial weakness in patients with RHS, despite the absence of high quality data [4, 19]. Rapid improvement of facial palsy after appropriate antiviral therapy is one of the interesting findings in this case report. Our patient had full recovery of her right facial palsy within 48 hours after initiation of acyclovir. However, as evidenced by our case, rapid improvement of facial palsy may not necessarily predict improvement of CNS findings. Vesicular lesions, diplopia, imbalance, and cerebellar symptoms did not improve until a tapering course of prednisone was administered.

Combination therapy of antivirals with corticosteroids has been observed by some investigators to result in improved outcomes such as the recovery of facial paralysis and facial nerve function [20], with the greatest benefit being observed with early treatment [16]. Recent reviews assessing the effectiveness of corticosteroids as adjuvant therapy to antivirals in restoration of facial nerve function in RHS have yielded conflicting results, indicating a need for randomized controlled trials [4, 21]. Follow-up brain MRI in our patient showed evidence of possible acute to subacute vasculitis. The effects of prolonged courses of antivirals or steroids on the vasculitic effects of VZV are unclear. Further investigations including repeat MRI and DSA would be helpful in this regard. The recurrence of right ear vesicular lesions in our patient may have been a complication of steroid therapy.

## Figures and Tables

**Table 1:** Cerebrospinal fluid (CSF) results.

Spinal fluid	Value	Reference range
Appearance	Cloudy and colourless	
Glucose	**5.2 mmol/L**	2.2–3.9 mmol/L
Protein	**669 mg/L**	200–400 mg/L
Nucleated cells	**700 × 10** ^**6**^ **/L**	0 × 10^6^/L–5 × 10^6^/L
Lymphocytes	**89%**	—
Neutrophils	1%	—
Monocytes	9%	—
Nonhemopoietin %	1%	—

Serology/microbiology/virology	Result	

Varicella zoster virus PCR	*Positive*	
Herpes simplex virus PCR	Negative	
Enterovirus PCR	Negative	
Arbovirus IgM	Nonreactive	
Bacterial culture	Negative	

PCR: polymerase chain reaction.
